# Mathematical Modeling of Interleukin-27 Induction of Anti-Tumor T Cells Response

**DOI:** 10.1371/journal.pone.0091844

**Published:** 2014-03-14

**Authors:** Kang-Ling Liao, Xue-Feng Bai, Avner Friedman

**Affiliations:** 1 Mathematical Biosciences Institute, The Ohio State University, Columbus, Ohio, United States of America; 2 Department of Pathology and Comprehensive Cancer Center, The Ohio State University, Columbus, Ohio, United States of America; 3 Department of Mathematics, The Ohio State University, Columbus, Ohio, United States of America; University of Arizona, United States of America

## Abstract

Interleukin-12 is a pro-inflammatory cytokine which promotes Th1 and cytotoxic T lymphocyte activities, such as Interferon-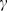 secretion. For this reason Interleukin-12 could be a powerful therapeutic agent for cancer treatment. However, Interleukin-12 is also excessively toxic. Interleukin-27 is an immunoregulatory cytokine from the Interleukin-12 family, but it is not as toxic as Interleukin-12. In recent years, Interleukin-27 has been considered as a potential anti-tumor agent. Recent experiments *in vitro* and *in vivo* have shown that cancer cells transfected with IL-27 activate CD8^+^ T cells to promote the secretion of anti-tumor cytokines Interleukin-10, although, at the same time, IL-27 inhibits the secretion of Interferon-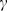 by CD8^+^ T cells. In the present paper we develop a mathematical model based on these experimental results. The model involves a dynamic network which includes tumor cells, CD8^+^ T cells and cytokines Interleukin-27, Interleukin-10 and Interferon-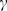. Simulations of the model show how Interleukin-27 promotes CD8^+^ T cells to secrete Interleukin-10 to inhibit tumor growth. On the other hand Interleukin-27 inhibits the secretion of Interferon-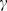 by CD8^+^ T cells which somewhat diminishes the inhibition of tumor growth. Our numerical results are in qualitative agreement with experimental data. We use the model to design protocols of IL-27 injections for the treatment of cancer and find that, for some special types of cancer, with a fixed total amount of drug, within a certain range, continuous injection has better efficacy than intermittent injections in reducing the tumor load while the treatment is ongoing, although the decrease in tumor load is only temporary.

## Introduction

Interleukin-12 (IL-12) is a pro-inflammatory cytokine that plays a central role in the connection of the innate resistance and adaptive immunity, by promoting Th1 and cytotoxic T lymphocyte (CTL) activities, such as IFN-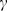 secretion. IL-12 could be a powerful therapeutic agent to eradicate tumor or to prevent the development of metastasis [1]–[4]. However, IL-12 has also been shown to be excessively toxic [5], [6], although there is at least one ongoing clinical trial with IL-12 using a new delivery method (IL-12 DNA plasmid) that is intended to overcome toxicity problems. In recent years there has been increasing interest to investigate the role of another member of the IL-12 family, namely, Interleukin-27 (IL-27), which is less toxic than IL-12, as a potential anti-tumor agent [7]. IL-27 is a cytokine capable of regulating Th1, Th2, Th17, and T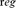 responses [8]. In autoimmune diseases, Murugaiyan et al. [9] have shown that IL-27 promotes production of IL-10 and IFN-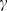 by naive human CD4^+^ T cells, and Stumhofer et al. [10] demonstrated that IL-27 promotes production of IL-10 by CD4^+^ and CD8^+^ T cells. Reviewing the role of IL-27 in anti-cancer immunotherapy, Swarbrick et al. [11] asserted that IL-27 may have both pro-inflammatory and anti-inflammatory functions, and it promotes Th1 immune response and CD8^+^ cell activation. Since Hisada et al. [7] first reported the anti-tumor efficacy of IL-27 in 2004, the potent anti-tumor activity of IL-27 has been verified in various tumor models [11]–[13]. Many studies suggest a role of IL-27 in enhancing anti-tumor CD8^+^ T cell responses [7], [14]–[17]. The enhancing role of IL-27 in generating anti-tumor CTL response was also demonstrated using IL-27R deficient mice [18], [19].

IL-10 has inhibitory and stimulatory effects on human CD8^+^ T cells [20], and in viral infection it is known to inhibit effector and memory CD4^+^ T cell responses but not memory CD8^+^ T cells [21]. IL-10 may have positive or negative effect on tumor suppression (Asadullah et al. [22]). Numerous studies (e.g. [23], [24]) show that increase in IL-10 produced by macrophages is associated with tumor progression, while other studies [25]–[28] suggest that IL-10 plays a positive role in tumor rejection. IL-27 can induce production of IL-10 in CD8^+^ T cells [10], [29]. In a recent study, Liu et al. [30] used P1CTL TCR transgenic mouse model and mouse plasmacytoma tumor system to investigate how IL-27 enhances the anti-tumor responses. They found that IL-27 significantly enhances the survival of activated tumor antigen specific CD8^+^ T cells *in vitro* and *in vivo*, and induces IL-10 upregulation in these T cells. It was also suggested in [30], and demonstrated in [25]–[28], that CTL IL-10 production contributes to tumor rejection. These results have important implications for designing IL-27-based immunotherapy against human cancer.

In the present paper, we develop a mathematical model that describes the anti-tumor activity of CD8^+^ T cells in terms of IFN-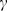 and IL-10 productions, when these T cells are activated by IL-27 from the tumor microenvironment. The model is based on the experiments by Liu et al. [30] (with mice infected with plasmacytoma) whereby cancer cells are transfected with an IL-27 vector so that IL-27 is released in the tumor microenvironment. We show that the model simulations agree qualitatively with the experimental results of [30]. We next extend the model to include therapeutic treatment of cancer in wild type mice by injection of IL-27. We note however, that in this case, the tumor microenvironment includes both CD4^+^ and CD8^+^ T cells (whereas in the experiments with transgenic mice of [30] the CD8^+^ T cells were taken to be dominant). As mentioned above, IL-27 promotes the secretion of IL-10 and IFN-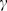 by CD4^+^ T cells [9]–[11], and we assume that these cytokines have the same effect on tumor rejection as those secreted by CD8^+^ T cells. We then use CD8^+^ T cells to represent both cells, CD4^+^ and CD8^+^. In the modified model the only source of IL-27 comes from the drug, since cancer cells do not generally secrete IL-27. We compare the efficacy of different strategies of IL-27 injections. For example, we found that continuous injection of IL-27 for 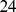 weeks at a fixed amount 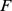, within a certain range, is more effective than intermittent injection of the amount 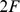, full three weeks at a time with three weeks spacing between injections, for 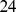 weeks. These predictions however must be viewed just as suggestions since they may only apply to special types of cancer, such as plasmacytoma in bone or soft tissue, and since, furthermore, the model does not include other important factors in tumor progression such as angiogenesis and the immune response.

## Results

### Mathematical model

**Figure 1:**
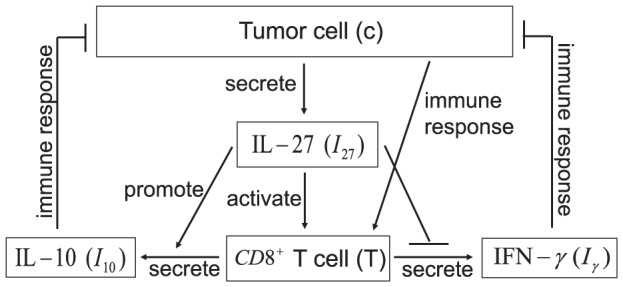
A network of IL-27. A network showing how IL-27 affects the immune response to tumor cells. CD8^+^ T cells are activated by P1A antigen from tumor cells as well as by IL-27 which is secreted by tumor cells. Activated CD8^+^ T cells secrete IFN-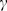, inhibited by IL-27, and IL-10 enhanced by IL-27. IL-10 and IFN-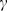 inhibit tumor cells.

In this model, we assume that the tumor is spherical and that it initially lies in a spherical tissue of radius 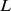. The variables that will be used in the model are listed below and we assume that all the variables are radially symmetric:
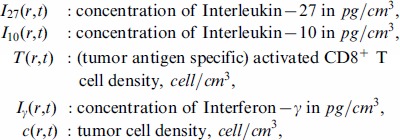
where 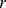 is the distance from a point 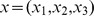 to the origin: 
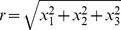
 and 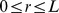. These variables satisfy a system of partial differential equations based on the network exhibited in Figure 1. The parameter values are estimated in Methods. In our model we shall include diffusion of cells and cytokines, as was done in many other models of solid tumors (which include plasmacytoma [31]–[38])

#### IL-27

The following equation describes the evolution of 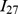:
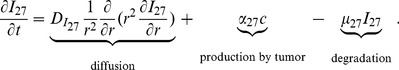
(1)


The first term represents the diffusion of 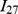 with coefficient 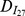. Although diffusivities of cytokines and cells may depend on the concentrations of the tumor cells and normal healthy cells, for simplicity, here and in the sequel all diffusivities are assumed to be spatially uniform. In the experiment in [30], Liu et al. used gene transfected tumor cells, J558-IL-27, to produce 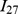 in the tumor microenvironment. Accordingly, we use the second term to describe the production of 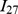 by the transfected J558-IL-27 tumor cells. The last term stands for the degradation of 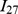. The parameter values of Equation (1) are given in Table 1.

**Table 1:** Parameters for the IL-27 equation.

Parameter	Description	Value with unit	Reference
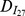	diffusion coefficient of 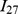	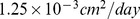	[48] & estimated
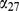	production rate of 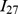 from tumor	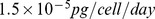	[30] & estimated
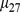	degradation rate of 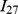	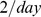	[48] & estimated

#### IL-10

The Interleukin-10 (IL-10) in Figure 1 is pro-inflammatory, in accordance with the experiments of [30]. It satisfies the equation:
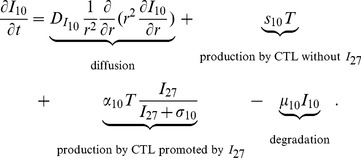
(2)


The first term is the diffusion of 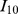. The second term accounts for the production of 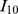 by CD8^+^ T cells for the absence of 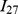 [30]. The experiments in [30] indicate that 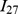 significantly increases the production of 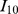 by CD8^+^ T cells, and this is accounted by the third term. The last term is the degradation of 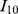. The parameter values of Equation (2) are listed in Table 2.

**Table 2:** Parameters for the IL-10 equation.

Parameter	Description	Value with unit	Reference
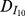	diffusion coefficient of 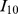	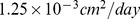	[48]
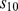	production rate from CTL without IL-27	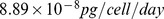	[30] & estimated
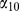	max production rate from CTL with IL-27	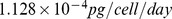	[30] & estimated
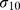		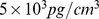	[49] & estimated
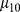	degradation rate of 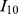	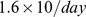	[48]

#### CD8^+^ T cells

The equation for the density of (activated) CD8^+^ T cells, 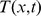, is given by
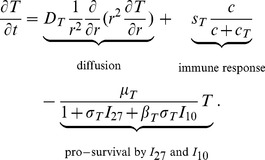
(3)


The first term is a dispersion of CD8^+^ T cells with coefficient 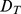. The second term accounts for activation of CD8^+^ T cells by P1A antigen from the tumor cells. 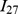 promotes survival of CD8^+^ T cells, and so does also 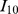, but to a smaller degree [30]. We present these two facts by correspondingly decreasing the death rate 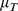 of T cells in the last term of Equation (3). The parameter values in Equation (3) are given in Table 3. Although 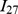 contributes more than 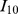 to promote the half-life of CD8^+^ T cells, we take 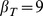 since the concentration of 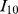 is much smaller than the concentration of 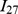.

**Table 3:** Parameters for CD8^+^ T cell equation.

Parameter	Description	Value with unit	Reference
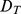	diffusion coefficient of CTL	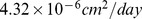	[48]
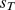	production rate of CTL activated by tumor	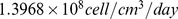	[30] & estimated
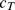		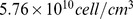	estimated
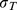		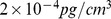	estimated
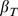		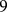	estimated
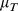	death rate of CTL	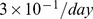	[48]

#### IFN-γ

Interferon-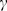 (IFN-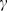) is a cytokine with diffusion coefficient 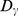 and degradation rate 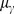. It is produced by T cells and, as shown in [30], the production is inhibited by 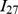. Thus, 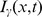 satisfies the equation:
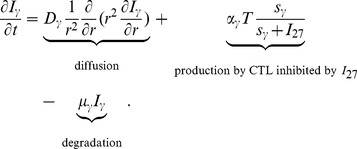
(4)


Table 4 lists all parameter values of (4).

**Table 4:** Parameters for the IFN-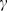 equation.

Parameter	Description	Value with unit	Reference
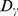	diffusion coefficient of 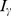	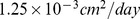	[48] & estimated
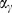	max production rate of 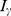 from CTL	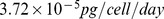	[30] & estimated
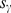		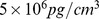	estimated
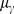	degradation rate of 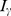	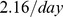	[49]

#### Tumor cells

The density of tumor cells, 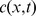, satisfies the following equation:
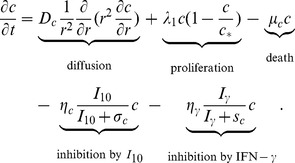
(5)


The second and third terms represent the proliferation and death of cells, respectively. Generally, 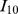 is regarded as an anti-inflammatory cytokine. However, in different experimental models, 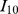 could suppress or promote the functions of immune system [30], [39]. Liu et al. [30] found that the 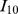 produced by CTL contributes to tumor rejection. Hence, the fourth term accounts for the indirect inhibition of tumor cells by 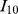. Cytokine, 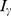 promotes the anti-tumor response, such as increase production of IL-12, and induces natural killer cells to kill cancer cells [40], [41]. For simplicity, we take the fifth term in (5) to represent the (indirect) inhibition of tumor cells by 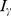. The parameter values are listed in Table 5.

**Table 5:** Parameters for tumor cell equation.

Parameter	Description	Value with unit	Reference
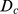	diffusion coefficient of tumor	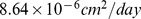	[48]
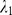	max proliferation rate	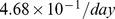	[30] & estimated
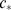		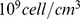	[48]
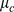	death rate of tumor	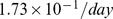	[48] & estimated
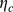	inhibition rate of tumor from 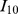	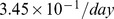	[30] & estimated
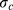		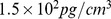	estimated
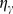	inhibition rate of tumor from IFN- 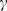	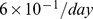	[30] & estimated
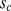		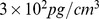	estimated

The dimensional and dimensionless values of all the parameters of Tables 1–5 are listed in Table 6.

**Table 6:** Model variables and units.

Parameter	Dimension value	Dimensionless value
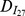	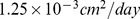	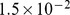
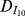	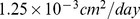	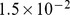
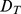	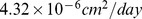	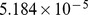
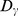	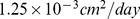	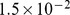
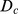	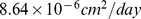	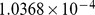
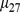	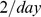	6
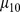	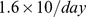	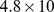
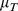	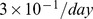	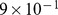
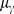	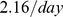	6.48
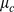	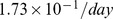	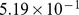
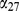	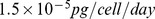 for small production	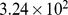
	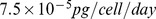 for moderate production	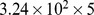
	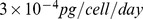 for large production	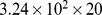
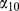	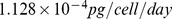	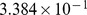
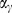	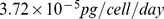	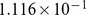
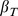	9	9
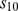	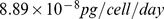	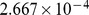
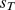	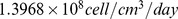	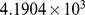
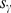	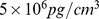	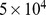
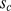	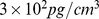	3
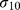	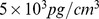	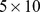
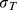	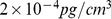	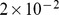
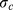	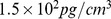	1.5
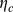	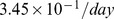	1.035
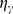	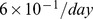	1.8
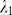	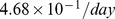	1.404
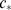	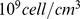	1.38889
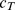	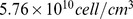	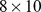
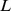	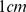 in Figs. 2–5	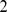
	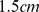 in Figs. 6–10	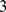
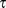	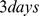	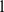
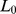	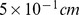	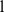
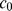	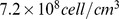	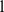
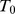	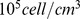	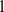
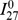	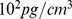	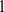
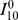	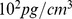	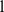
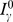	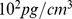	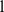
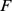	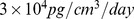 in Figs. 6, 7, and 10	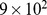
	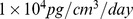 in Figs. 8A and 9A	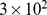
	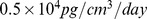 in Figs. 8B and 9B	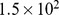
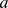	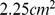	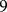

#### Initial conditions

We assume that tumor cells are initially concentrated near 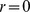, taking
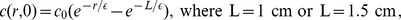
and 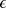 is a positive number less than or equal to 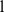. In the simulations, we shall take 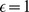 but the results do not change qualitatively with smaller values of 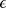. Since 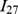 is produced by J558-IL-27 tumor cells, the initial concentration of 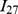 should be similar to the density of tumor cells; we take
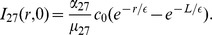
Initially, there are no activated CD8^+^ T cells, hence
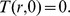



Since 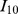 and 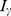 are produced by CD8^+^ T cells, we take
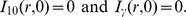



#### Boundary conditions

Since all variables are radially symmetric, the first 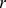-derivative at 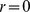 is equal to zero. We assume no-flux condition for all variables at 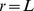. This is justified by the fact that 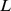 is large enough so that the exterior of the ball of radius 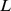 lies completely within the healthy tissue, initially.

#### Parameters nondimensionalization

We nondimensionalizate the Equations (1) – (5):
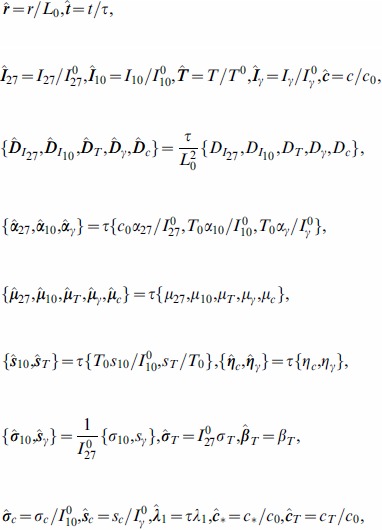
where
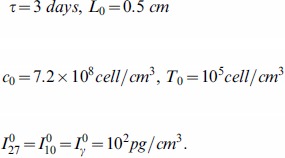



For nondimensional variables and parameters, we consider the tumor growth in a ball 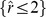 or 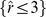. The nondimensional PDE model is given by the following system of equations:
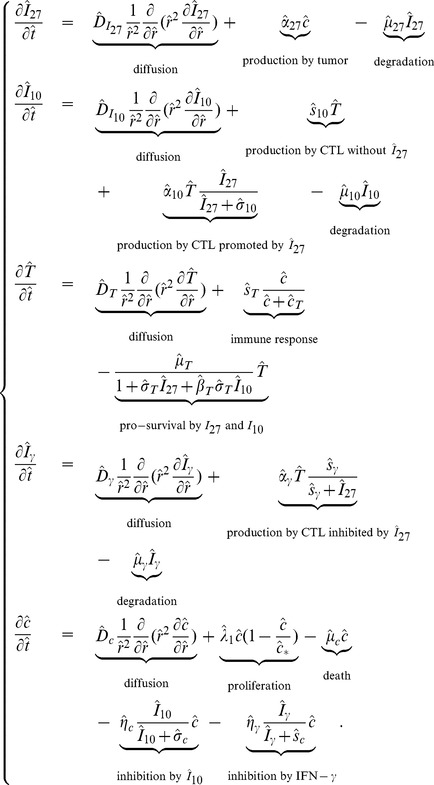
(6)


### Numerical simulation

The model (6) was simulated, in nondimensional variables, using matlab with 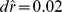 and 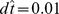 (i.e., 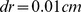 and 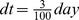 in dimensional units). Four cases were considered:

- J558-Ctrl tumor cells.
- J558-IL-27 tumor cells with small production rate of IL-27.
- J558-IL-27 tumor cells with moderate production rate of IL-27.
- J558-IL-27 tumor cells with large production rate of IL-27.

It has been reported in [30] that 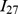 can enhance the population of CD8^+^ T cells. Moreover, 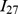 also enhances 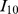 produced by CD8^+^ T cells to inhibit the tumor growth, but at the same time it suppresses the pro-inflammatory cytokine 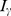 secreted by CD8^+^ T cells. In spite of its inhibition of 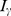, 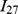 still promotes CD8^+^ T cells to suppress the tumor growth.

In view of these experimental results we expect the total mass of 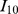 to increase from cases (i) to (iv), the total CD8^+^ T cell population to increase from cases (i) to (iv), and the total population of cancer cells to decrease from cases (i) to (iv), as time progresses.

**Figure 2:**
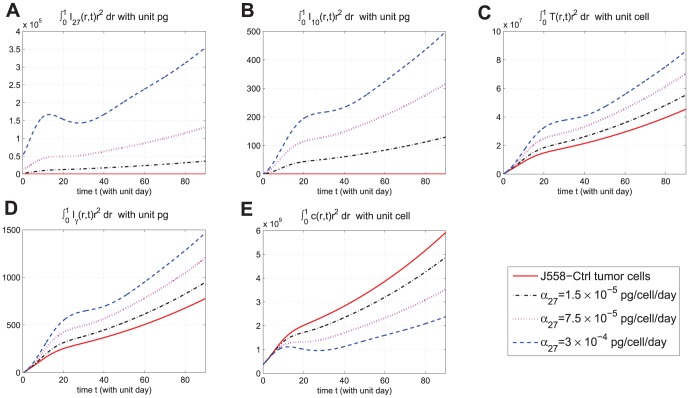
Evolution of cells and cytokines for different production rates of IL-27. (A), (B), (C), (D), and (E) are the profiles of total number of 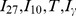, and 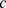, respectively, within 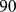 days. In (E), the curves displayed from top to bottom are for J558-Ctrl tumor cells, J558-IL-27 tumor cells with small (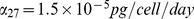), moderate (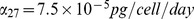), and large (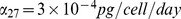) production of IL-27, successively; 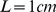.

**Figure 3:**
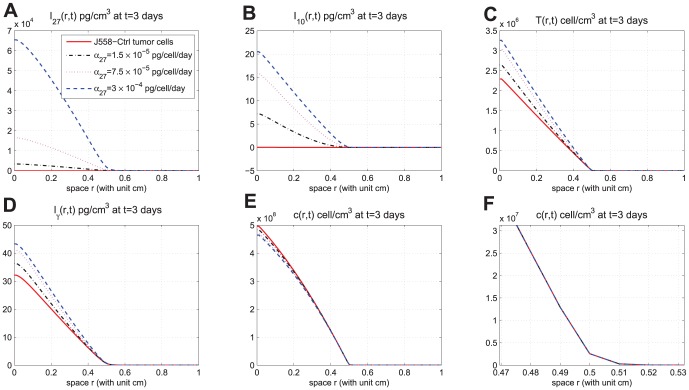
Spatial distributions at day 3. (A), (B), (C), (D), and (E) are the spatial distributions of 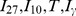, and 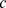, respectively, at day 3 for different production rates of IL-27. (F) is zoomed in (E) near 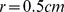. In (E), the curves displayed from top to bottom are for J558-Ctrl tumor cells, J558-IL-27 tumor cells with small (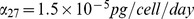), moderate (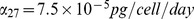), and large (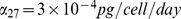) production of IL-27, successively; 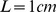.

**Figure 4:**
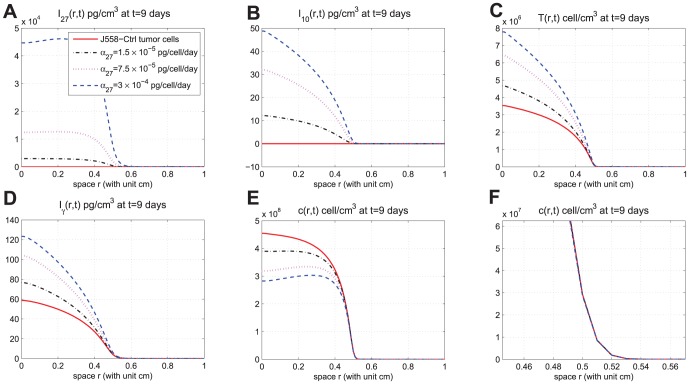
Spatial distributions at day 9. (A), (B), (C), (D), and (E) are the spatial distributions of 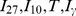, and 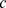, respectively, at day 9 for different production rates of IL-27. (F) is zoomed in (E) near 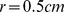. In (E), the curves displayed from top to bottom are for J558-Ctrl tumor cells, J558-IL-27 tumor cells with small (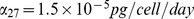), moderate (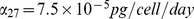), and large (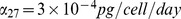) production of IL-27, successively; 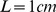.

**Figure 5:**
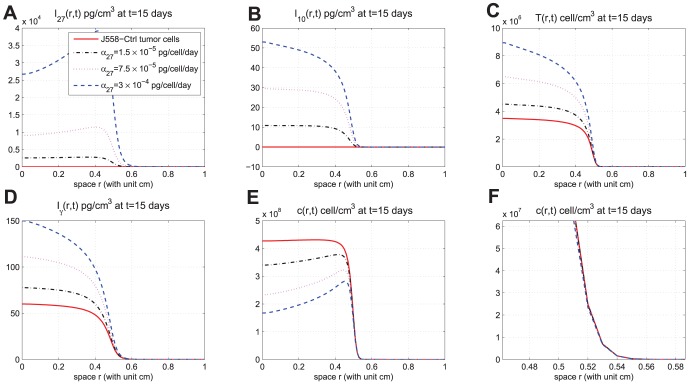
Spatial distributions at day 15. (A), (B), (C), (D), and (E) are the spatial distributions of 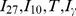, and 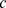, respectively, at day 15 for different production rates of IL-27. (F) is zoomed in (E) near 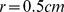. In (E), the curves displayed from top to bottom are for J558-Ctrl tumor cells, J558-IL-27 tumor cells with small (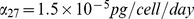), moderate (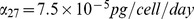), and large (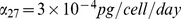) production of IL-27, successively; 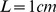.

Correspondingly, we associate with the four cases (i) – (iv) increasing values of the parameter 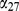:
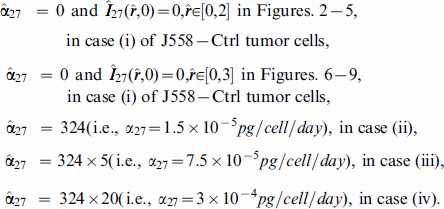



In Figures 2-5, we took 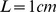 such that tumor cells are not visible near the boundary 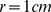 for all time 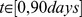. Figure 2 shows the time-dependent profiles of the total mass of 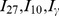, and total populations of CD8^+^ T cells and cancer cells. We see that growth/decrease of these variables, as 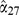 varies, corresponds qualitatively to the experiments in [30]. Figures 3-5 show significant spatial variations of these variables at days 3,9, and 15, with or without production of 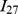. We also see the effect of 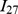 on cancer cells and CD8^+^ T cells densities at different distances from the point of origin of the cancer. For example, at the origin, at day 3 the cancer cells density changed from 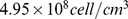 with no treatment by 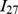 to 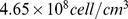 with largest production of 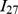, while at day 15 it changed from 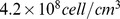 with no treatment to 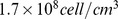 with largest production of 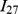. Similarly, at the origin, the CD8^+^ T cell density increased at day 3 from 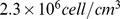 without treatment to 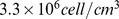 with largest production of 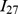, whereas at day 15 the density increased even more significantly from 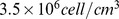 with no 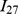 treatment to 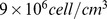 with maximal production of 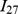. Note that Figure 5E and 5F show that the tumor cell density is almost zero near the boundary 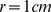 and the tumor cells concentrate in the region 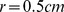.

Tumor initiating in internal organs can also be treated by 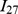, but the mechanism for introducing 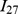 will depend on the location of the tumor. For example, in colitis induced colon cancer, one could use yeast which were programmed to express 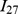 [42]. Oncolytic virus which are engineered to produce 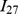 within tumor cells could turn the tumor into immunogenetic, thus enabling the immune system to reject the tumor.

We want to use our model in order to design treatments for a wild type mouse by 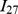 injection. We recall, as noted in the Introduction, that for wild type mouse, both CD4^+^ and CD8^+^ T cells produce IL-10 and IFN-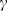 [9]–[11] and we assume that IL-10 secreted by CD4^+^ T cells has the same tumor rejection quality as the IL-10 secreted by CD8^+^ T cells. We then use CD8^+^ T cells to represent both cells, CD4^+^ and CD8^+^. We also note that *in vivo* tumor cells do not generally secrete 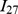, so we take 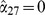 in Equation (1). But we also need to include an injection term in Equation (1) for 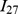. If we denote the injection density by 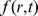 then Equation (1) becomes
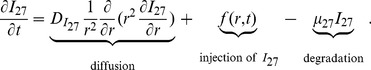
(7)


We make the pharmacokinetic assumption that 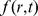 decreases in 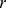 from the outer boundary of the tumor (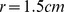) towards the inner core (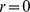), and take
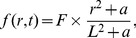
(8)where 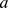 is some positive constant; 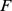 is viewed as the “amount” of injection.

We consider here, for illustration, two strategies of treatment: (i) continuous injection of 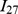 at a fixed amount 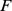 for 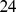 weeks, and (ii) intermittent injections, at double amount 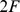, full three weeks at a time with three weeks spacing between injections. Accordingly, for the continuous strategy
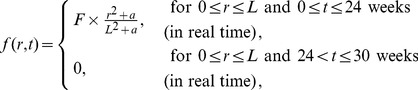
(9)and for the intermittent strategy
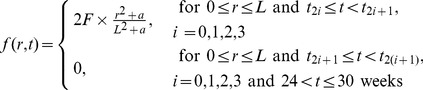
(10)in case (ii), where the length of each interval 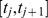 is three weeks (the drug is injected only during the intermittent intervals 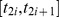) and 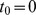. In the following simulations, we take 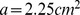; however the same results remain qualitatively the same for other values of 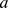 (not shown here).

**Figure 6:**
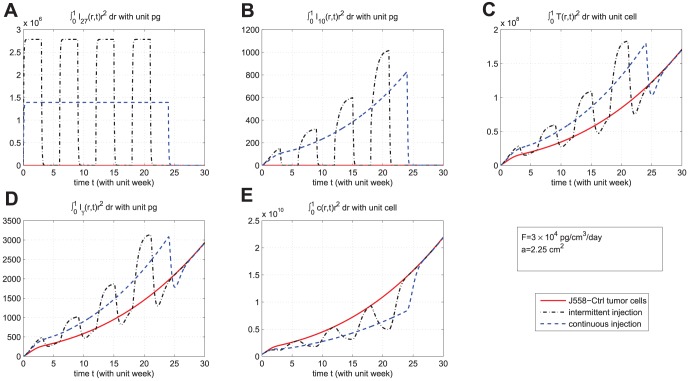
Comparison of continuous versus intermittent treatment. (A), (B), (C), (D), and (E) are the profiles of total number of 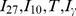, and 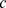, respectively, for model (6) with 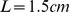 which the first equation for 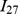 is replaced by (7) and all parameter values are taken from Table 6. In (E), the upper curve is for J558-Ctrl tumor cells, the dotted-dashed curve (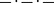) is for intermittent injection, and the dashed curve is for continuous injection with 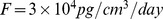 and 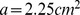, for the first 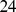 weeks.

**Figure 7:**
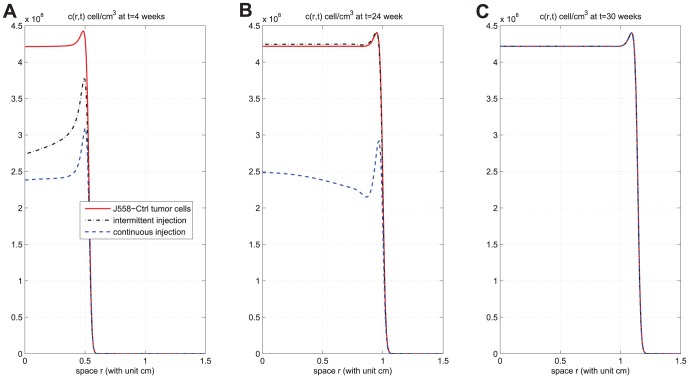
Concentration profiles of tumor cells at different times. (A), (B), and (C) are the concentration profiles of 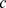 at times 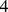 weeks (short time), 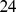 weeks (time at which injections are withdrew), and 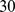 weeks (the final time for simulation), respectively, under drug amount 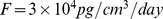 and 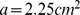. The upper curve is for J558-Ctrl tumor cells, the dotted-dashed curve (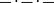) is for intermittent injection, and the dashed curve is for continuous injection. The concentration of tumor cells are not visible, when 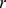 is close to 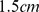, for all 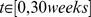; 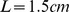.

**Figure 8:**
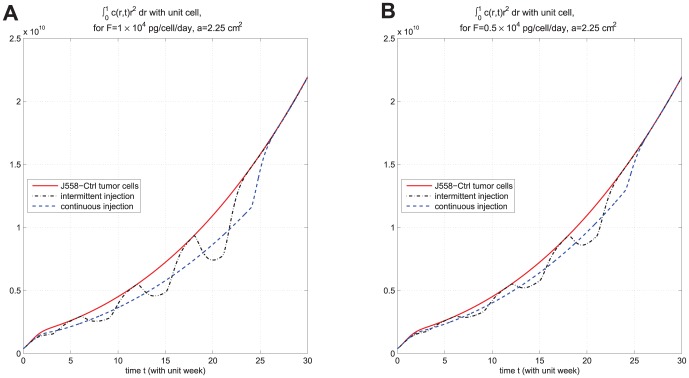
Comparison of continuous versus intermittent treatment for different drug amount. (A) and (B) are the profiles of total number of 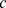 with 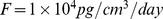 and 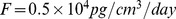, respectively, for model (6) with 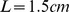 and 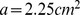 which the first equation for 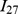 is replaced by (7) and all parameter values are taken from Table 6. The upper curve is for J558-Ctrl tumor cells, the dotted-dashed curve (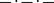) is for intermittent injection, and the dashed curve is for continuous injection.

**Figure 9:**
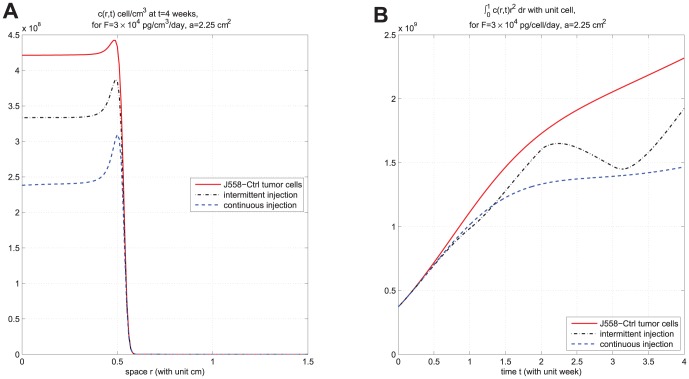
Tumor growth and migration for shorter injection schedule. (A) is the concentration profile of 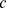 at 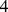 weeks and (B) is the profile of total number of 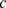 for 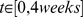, under 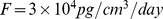, for model (6) with 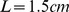 and 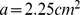 which the first equation for 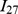 is replaced by (7) and all parameter values are taken from Table 6. The upper curve is for J558-Ctrl tumor cells, the dotted-dashed curve (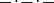) is for intermittent injection, and the dashed curve is for continuous injection. (A) shows that the concentration of tumor cells are not visible near the boundary 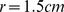, for all 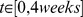.

In Figures 6-9, we take 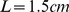 so that the tumor cell density remains negligible near the boundary 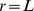, during the entire simulation time which is 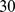 weeks and hence the boundary conditions are not affecting the results during the entire simulation (For longer simulation time, e.g. 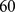 weeks, we need to take 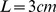 (not shown here.)). We also take the simulation mesh size 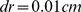 and 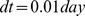. In Figure 6, we compare the results of the two strategies in case 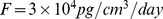. We see that continuous injection yields better results in reducing the tumor level and slightly delaying relapse after the drug is withdrawn. Figure 7, for the same experiment as in Figure 6, shows the concentration profiles of tumor cells at times 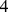 weeks, 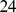 weeks, and 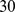 weeks for J558-Ctrl, intermittent injection, and continuous injection cases. Notice from Figures 7A, 7B, and 7C that the tumor has progressed during the periods of 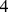 weeks, 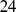 weeks, and 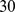 weeks to 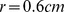, 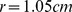, and 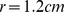, respectively. Figures 6 and 7 show that 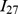 injection slows down tumor growth during drug injection, but it does not change the migration speed of tumor cells. Figure 8 compares the results of the above two strategies for smaller values of 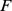, namely, 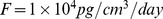 and 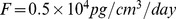. We see that continuous injection is still more effective, but, for smaller amount of injection, the relative advantage of continuous injection is decreased. Simulations of these two strategies for other values of 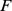 in the range of 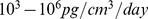 (not shown here) give the same results, namely, that continuous injection is preferable to intermittent injections. In order to make a definite recommendation on continuous versus intermittent injection one would need to consider also possible side-effects that may arise from these two strategies.

Although the expected lifespan of the mouse in the experiments of Liu et al. [30] was one month, for the purpose of therapy we performed simulations for the longer period of 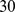 weeks. But it is also interesting to consider the case of treatment for one month only. This is done in Figure 9 where we have taken in Equation (9) 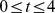 weeks for the continuous treatment, and, in Equation (10), intermittent time 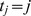 weeks. Figure 9 shows that continuous treatment is again preferable to intermittent treatment. Figure 9A shows the concentration profile of tumor cells at 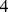 weeks; note that tumor cells do not reach the boundary within 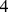 weeks. Figure 9B displays the profile of total number of tumor cells. We see that the continuous treatment still has better efficacy than intermittent treatment.

### Sensitivity analysis

In order to provide support to the robustness of the simulation results, we ran sensitivity analysis on parameters which appear in Equations (1) – (5). The parameters chosen for the sensitivity analysis are either those whose baseline was crudely estimated, or those that seem to play more important role in the model predictions.

We list these parameters with their ranges, baselines, and units, in Table 7. In this analysis, 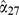 varies from 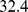 to 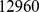. Following the sensitivity analysis method described in [43], we performed Latin hypercube sampling and generated 5000 samples to calculate the partial rank correlation coefficients (PRCC) and p-value, with respect to the ratio 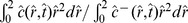, where 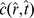 (resp. 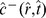) accounts for the J558-IL27 (resp. J558-Ctrl) tumor cell density, at 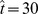 and 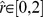. The PRCC and their p-values are listed in Table 8. A negative PRCC (i.e. negative correlation) means increase in the parameter value will decrease the ratio 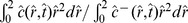; that is, it will increase the rejection of tumor treated by IL-27 versus untreated tumor. Conversely, positive PRCC means that increased rejection of the tumor (treated by IL-27 versus untreated) will occur if this parameter is decreased.

**Table 7:** Parameters chosen for sensitivity analysis.

Parameter	Range	Baseline	Unit
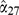	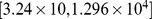	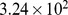	pg/cell/day
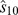	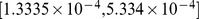	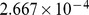	pg/cell/day
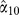	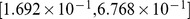	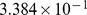	pg/cell/day
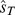	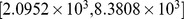	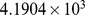	cell/cm^3^/day
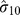	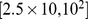	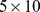	pg/cm^3^
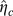	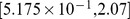	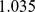	/day
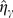	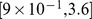	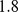	/day
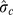	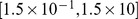	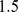	pg/cm^3^
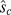	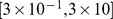	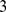	pg/cm^3^
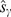	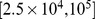	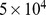	pg/cm^3^
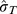	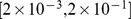	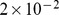	pg/cm^3^
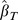	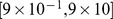	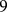	nondimension
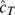	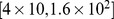	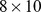	cell/cm^3^

**Table 8:** The PRCC and p-value of parameters for sensitivity analysis.

Parameter	PRCC	p-value
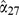	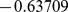	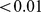
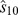	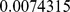	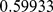
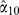	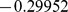	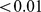
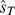	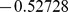	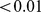
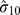	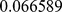	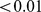
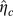	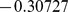	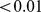
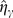	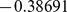	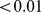
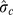	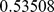	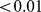
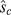	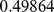	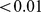
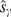	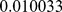	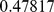
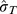	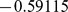	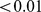
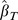	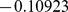	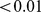
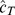	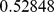	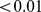

The sensitivity analysis data are shown in Figures S1–S4 in Supplementary Material and summarized in Table 8. The most significant negatively correlated parameters in promoting rejection of tumor treated by IL-27 versus untreated tumor are 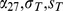; less significant parameters are 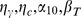. The effect of 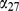 has already been displayed in Figures 2–5. The negative correlations of 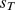, 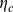, and 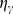 are not surprising, since 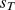 is the rate by which tumor activates T cells (while T cells are increased with IL-27 treatment; see Figures 2–5) and 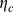 and 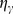 are, respectively, the killing rates of tumor cells by 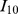 and 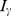 (while 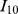 and 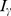 increase with IL-27 treatment; see Figures 2–5). The negative correlations of 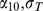, and 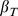 are also not surprising, since 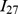 promotes the production of 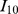 to inhibit tumor cells, and larger 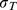 and 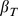 promote survival of CD8^+^ T cells.

The most significant parameters in promoting tumor are 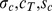 and, to a smaller degree, 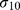. This also is not surprising, since increasing 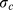 results in decreased inhibition of tumor cells by 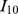, increasing 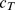 results in decreased number of CD8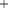 T cells, increasing 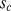 results in decreased inhibition of tumor cells by 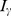, and increasing 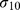 results in decreased 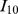. We note that the parameters 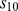 and 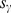, in Table 8, have small PRCC with p-values that are larger than 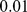; this means that they are not sensitive to the ratio 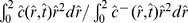.

## Discussion

IL-12 plays a central role in linking the innate resistance and adaptive immunity, and could be a powerful anti-tumor agent. However, since IL-12 is excessively toxic, the cytokine IL-27, which is a less toxic member of the IL-12 family, has been considered as a possible replacement of IL-12 as anti-tumor agent [7], [8], [14]–[17]. It was demonstrated by Liu et al. [30] that IL-27 enhances the survival of tumor antigen specific CD8^+^ T cells and induces their upregulation of IL-10, which acts as an anti-tumor cytokine. This suggests that IL-27 could play an important role in immunotherapy against human cancer.

The aim of the present paper was to develop a mathematical model that can be used to explore and predict the efficacy of different protocols of IL-27 treatment. To do that we first set up a dynamical system of partial differential equations whereby IL-27 is produced by transfected J558-IL-27 tumor cells, as demonstrated in the experiments of Liu et al. [30]. The model included IL-27-induced CD8^+^ T cells and cytokines IL-10 and IFN-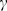. By carefully estimating the parameters of the equations we showed that the model simulations agree with the experimental data of Liu et al. [30].

The model can be used to examine the effect of injecting IL-27 into the microenvironment of cancer in a mouse, and design strategies for such injections. We illustrated this by comparing the efficacy of two protocols: (i) continuous injection (e.g., daily) of IL-27 for 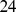 weeks at a fixed amount 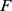, and (ii) intermittent injections during the first 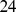 weeks with three weeks injection at a fixed amount 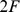 followed by three weeks spacing, and withdrawing the drug after the 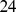 weeks for both protocols (i) and (ii). We found that the continuous injection has better efficacy in reducing the tumor load, and also in delaying relapse after the drug is withdrawn, while the treatment is ongoing. However, in establishing these results we made the assumption that IL-10 produced by IL-27 activated CD4^+^ T cells has the same pro-inflammatory property as the IL-10 produced by CD8^+^ T cells. In addition, we made the pharmacokinetic assumption that the drug density decreases toward the inner core of the tumor, and we also took the drug “amount” 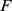 in the range of 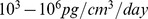.

We note that our model was based on the experiments by Liu et al. [30] with plasmacytoma, but not with other tumor cells. Furthermore, the model did not include the effects of lymphoid and vascular compartments, as these were not reported in [30]. Hence the present paper should be considered only as an initial building block for a more comprehensive model which should include angiogenesis as well as the immune response of macrophages, dendritic cells, and T cells (Th1, Th2, Th17, and T
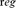
s). We note in particular that pro-inflammatory macrophages (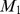) secrete a family of IL-12 cytokines including IL-27 [44], and the IL-12 family attracts CTLs which kill tumor cells, so that 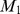 macrophages suppress tumor growth. On the other hand, anti-inflammatory macrophages (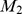) secrete IL-10 which promotes tumor growth [23], [24]. Regulatory T cells promote tumor growth and are inhibited by IL-27 [45], [46]. Thus the present paper's prediction of the efficacy of different protocols of treatment of plasmacytoma in bone or soft tissue with IL-27 will need to be re-examined when more data become available that will enable us to include the important compartments of the immune and vascular systems.

We note also that the proposed intervention with IL-27 in our paper shows benefits only while the treatment is ongoing. The treatment has neither significant short-term benefits nor any long-term benefits after the drug has discontinued. It is becoming increasingly common to treat tumors with several drugs. In addition to tumor specific drugs, a generic mitotic inhibitory drug, which disrupts microtubules that pull the cell apart, is often used – since cancer cells are more sensitive to inhibition of mitosis than normal healthy cells. In our model, the effect of such a drug is to increase the death rate parameter in the equation for cancer cells. Further work should also include the combined effect of treatment of IL-27 with a mitotic inhibitory drugs.

## Methods

### Estimates of the densities of tumor cells and T cells

Many of the parameters are based on experiments reported in [30]. In [30], the volume of the tumor was measured in days 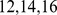, and 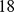, but the number of CD8^+^ T cells and concentrations of 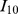 and 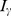 were measured only in the first 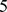 days.

From Figure 5D in [30], the volume of the tumor at days 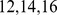, and 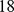 were approximate 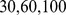, and 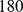 in 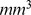. Hence,
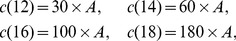
(11)where 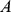 is the number of tumor cells in per 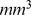. If we consider a simplified equation for (5)
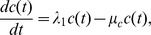
then, for any two times 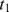 and 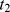,
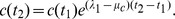



If we apply this formula to the 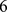 pairs of the numbers from (11) to compute 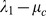 and take the mean value, we get
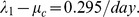



Since the half-life of melanoma tumor cells is approximate 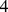 days, [47],
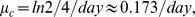
and then 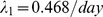.

In the experiments in [30], there were two kinds of tumor cells: J558-IL-27 which generates IL-27, and J558-Ctrl which does not generate IL-27. The antigen P1A on J558 tumor cells is recognized by receptors TCRs on cytotoxic T cells, P1CTL. Liu et al. [30] used P1CTL with glycoprotein CD8 (which is called CD8^+^ T cells) to investigate the immune response for IL-27. Their P1CTL cells were of four different types: (i) P1CTL which can recognize J558-Ctrl tumor cells and generate IL-10 to inhibit tumor growth; (ii) IL-10^-/-^P1CTL which can recognize J558-Ctrl tumor cells but cannot generate IL-10; (iii) P1CTL/IL-27 which can recognize J558-IL-27 tumor cells and generate IL-10; and (iv) IL-10^-/-^P1CTL/IL-27 which can recognize J558-IL-27 tumor cells but cannot generate IL-10.

The number of tumor cells at 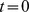 day (in [30]) was 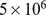 cells, and we assume (see Figure 5D in [30]) that they occupy volume 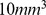. Hence
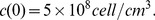
(12)


There is no data in [30] on the density of the tumor in day 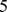. We assume that this density is larger than 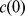 but substantively smaller than the maximal capacity 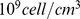. We take
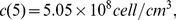
(13)for J558-Ctrl with P1CTL or J558-IL-27 with P1CTL/IL-27, but
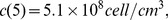
(14)for J558-Ctrl with IL-10^-/-^P1CTL or J558-IL-27 with IL-10^-/-^P1CTL/IL-27, since the last two types of T cells do not generate 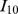.

From Figure 1A in [30], there were 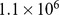 P1CTL at day 1 and 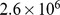 P1CTL at day 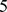; 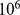 P1CTL/IL-27 at day 1 and 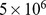 P1CTL/IL-27 at day 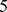. We assume that these CD8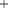 T cells occupy the volume of 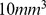 for the first 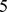 days. Hence
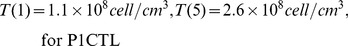
(15)




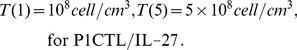
(16)


### Estimate of the parameters in (1)

Since IL-27 belongs to the IL-12 family, we take its diffusion coefficient and the degradation rate to be the same as for IL-12 [48]:
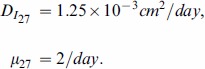



In order to find 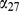, we use the simplified version of Equation (1):
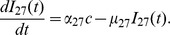



If 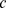 is taken to be a constant, then
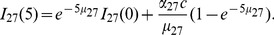
(17)


From Figure 1A in [30], 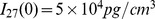 and only 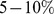 of 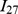 remained in day 5. We assume that 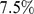 of 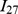 remained at day 5, so that
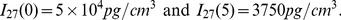
(18)


Taking 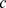 to be the average between the values at days 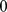 and 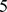 (see (12) and (13)) and recalling (17), we get
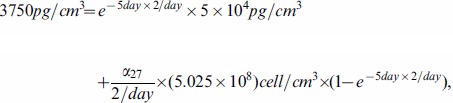



so that 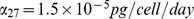.

### Estimate of the parameters in (2)

We consider a simplified version of Equation (2):
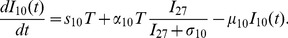
(19)


From [48], we have 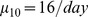. To estimate 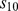, we consider the case of J558-Ctrl tumor cells, for which the term with 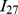 is removed from (19):
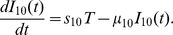



If 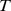 is constant, then
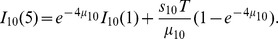



From the profile of P1CTL in Figure 3D of [30], we have 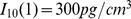 at day 1 and 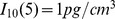 at day 5 and we take 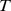 to be the mean value of 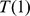 and 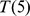 in (15). We then get
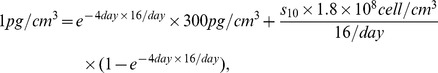
so that 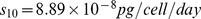.

Next, we choose 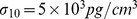 and proceed to compute 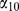. We then consider J558-IL-27 tumor cells which can generate 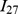. For simplicity, we take 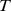 to be the average between the values at days 1 and 5 (see (16)) and 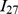 to be the average between the values at days 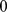 and 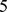 (see (18):
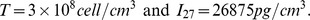



Then, the solution of Equation (19) satisfies
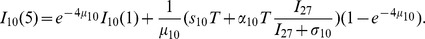



From the profile of P1CTL/IL-27 in Figure 3D of [30], we have 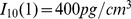 at day 1 and 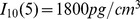 at day 5, so that
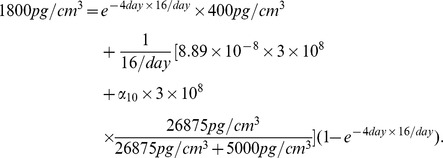



Therefore, we take 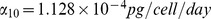.

### Estimate of the parameters in (3)

From [48], we have 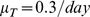. For J558-Ctrl tumor cells, the term of 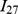 in (3) drops out, and we consider a simplified version:
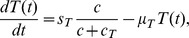
which, if 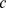 is constant, has the solution
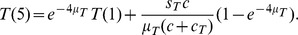



Substituting 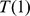 and 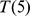 from (15), we get
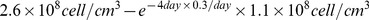
(20)




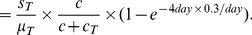
(21)


Based on the fact that the (20) is close to 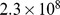 while the (16) is close to 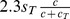, we choose 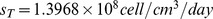.

Next, we consider
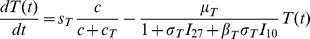
where the solution satisfies
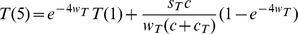
with 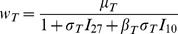. Recalling 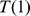 and 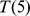 from (16), we get
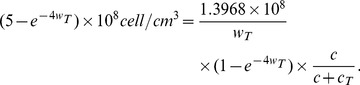
(22)


In (22), the left-hand side is close to 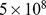 and the right-hand side is close to 
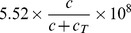
, while we take 
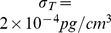
 and 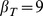.

Estimate of the parameters in (4)

We assume that the diffusion coefficient of 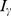 is the same as that of 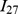, namely, 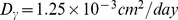. Next we use the simplified version:
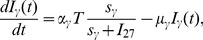
(23)


where 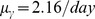 by [49]. For tumor cells J558-Ctrl (which do not generate 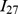), (23) reduces to
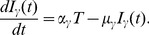
(24)


If 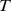 is constant, then the solution of (24) satisfies
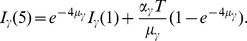



Taking 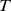 to be the average of 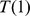 and 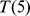 in (15) and taking 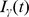 from the curve P1CTL in the right part of Figure 3D in [30], we have
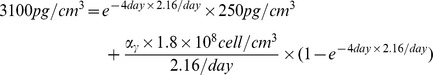



so that 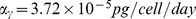. We choose 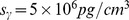.

### Estimate of the parameters in (5)

We consider a simplified version of (5):
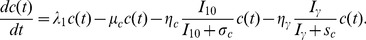
(25)


We choose 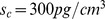. In order to compute 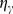, we consider T cells IL-10
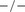
P1CTL and IL-10
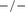
P1CTL/IL-27 which do not generate 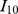, so that 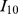 drops out of Equation (25):
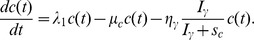



If 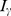 is constant, then the solution is 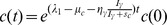 which leads to
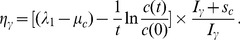
(26)


Since 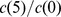 is close to 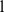 and the range of 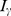 may vary from 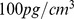 to 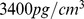 forJ558-Ctrl tumor cells and IL-10
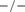
P1CTL T cells or from 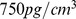 to 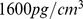 for J558-IL27 tumor cells and IL-10
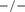
P1CTL/IL27, we choose in Equation (26)
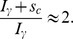



Recalling that 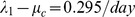, we take 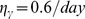.

Next, we choose 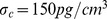 and proceed to estimate 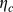 by considering T cells P1CTL and P1CTL/IL-27 which generate 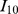. For (25), if 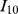 and 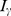 are constants, then the solution is 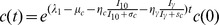, and hence
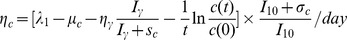


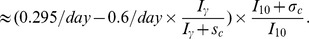



The concentration of 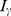 with 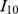 is smaller than the concentration of 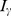 where 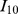 is blocked [30]. We take 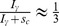. In [30], the concentration of 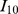 vary from 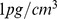 to 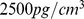. We take 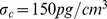, so that 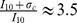; hence 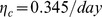.

## Supporting Information

Figure S1
**Sensitivity analysis.** Sensitivity analysis on 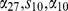, and 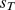.(PDF)

Figure S2
**Sensitivity analysis.** Sensitivity analysis on 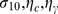, and 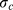.(PDF)

Figure S3
**Sensitivity analysis.** Sensitivity analysis on 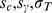, and 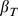.(PDF)

Figure S4
**Sensitivity analysis.** Sensitivity analysis on 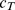.(PDF)
