# Supplementary figures and images for: Mathematical Modeling of Interleukin-27 Induction of Anti-Tumor T Cells Response
Source: PLoS One. 2014 Mar 14;9(3):e91844. doi: 10.1371/journal.pone.0091844 (PMC3954918; doi:10.1371/journal.pone.0091844)

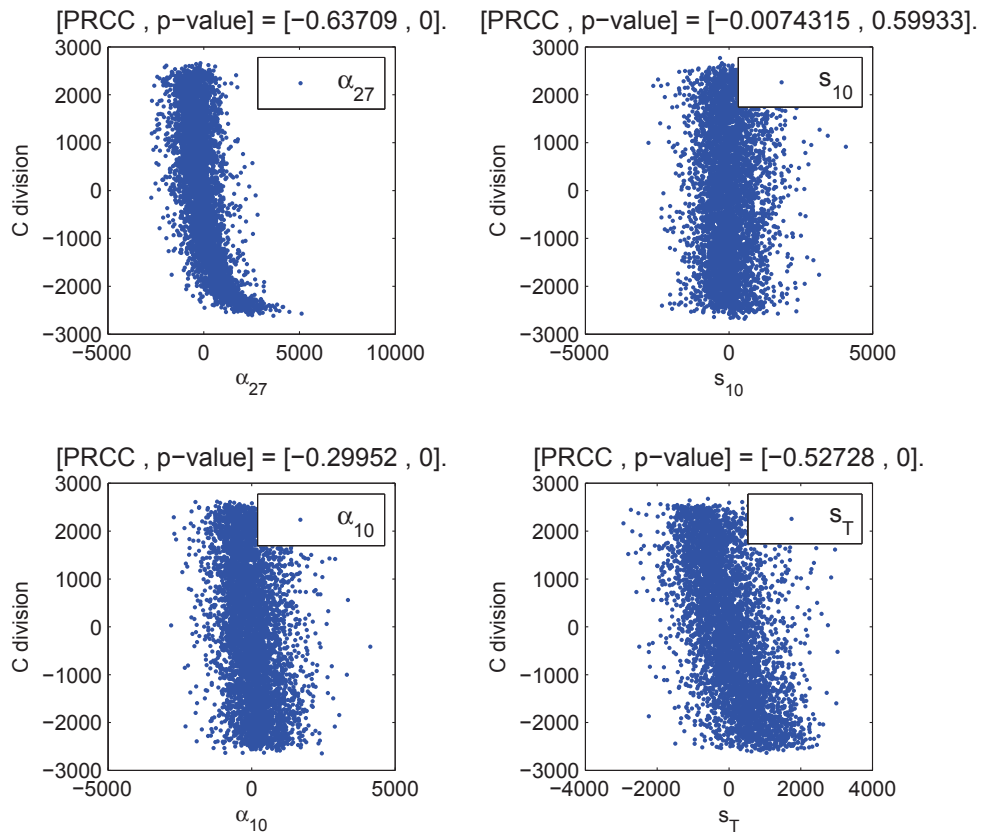

Supplement: Figure S1 — Sensitivity analysis. Sensitivity analysis on , and . (PDF) [file pone.0091844.s001.pdf]

[PRCC , p-value] = [0.066589 , 2.4459e-006]. [PRCC , p-value] = [-0.30727 , 0].

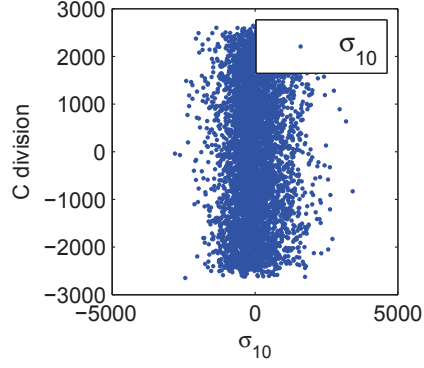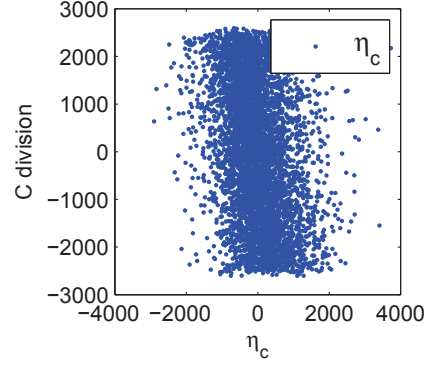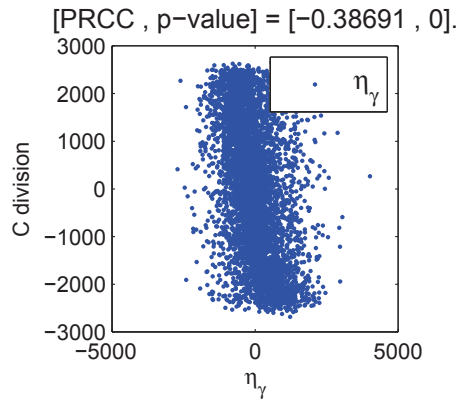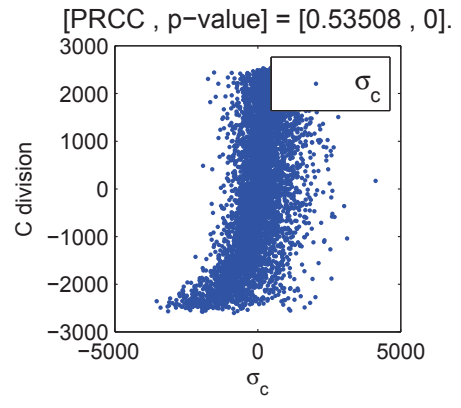

Supplement: Figure S2 — Sensitivity analysis. Sensitivity analysis on , and . (PDF) [file pone.0091844.s002.pdf]

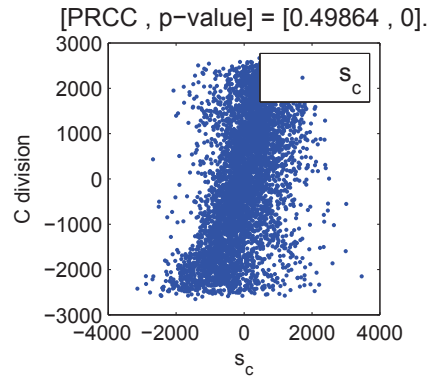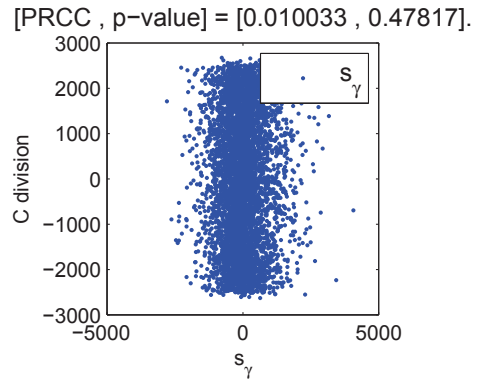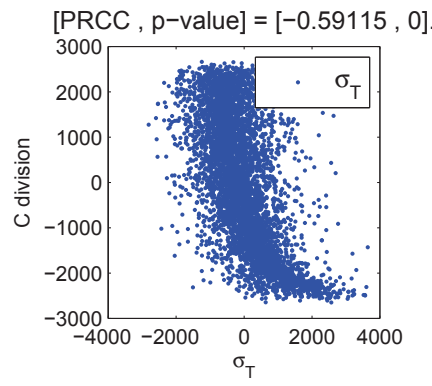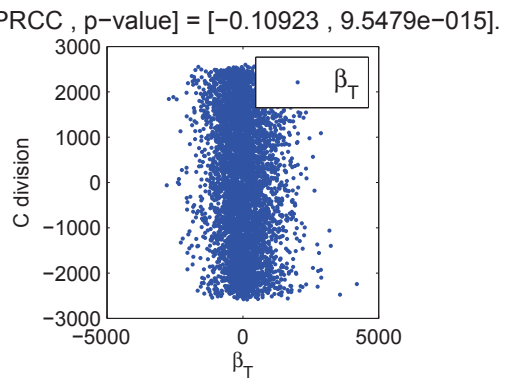

Supplement: Figure S3 — Sensitivity analysis. Sensitivity analysis on , and . (PDF) [file pone.0091844.s003.pdf]

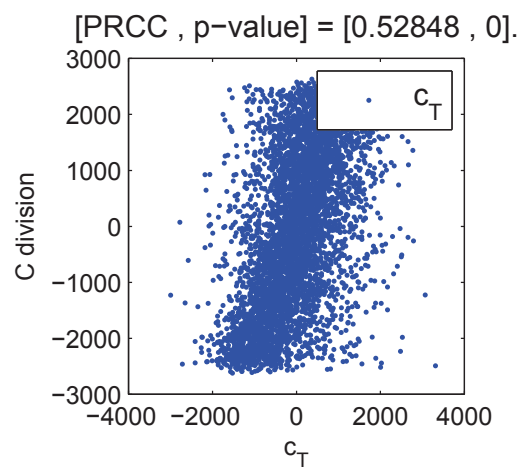

Supplement: Figure S4 — Sensitivity analysis. Sensitivity analysis on . (PDF) [file pone.0091844.s004.pdf]
